# Teachers’ Voice-Related Quality of Life in Relation to Environmental Noise in Schools: A Multidimensional Study Using VHI Test and Listen Responsibly App

**DOI:** 10.3390/audiolres15050138

**Published:** 2025-10-13

**Authors:** Jessica Frangipane, Pasquale Viola, Roberto Minici, Alfonso Scarpa, Alessia Astorina, Teodoro Aragona, Emilio Avallone, Federico Maria Gioacchini, Pietro De Luca, Giampietro Ricci, Valeria Gambacorta, Eva Orzan, Giuseppe Chiarella

**Affiliations:** 1 Unit of Audiology, Regional Centre for Cochlear Implants and ENT Diseases, Dulbecco University Hospital, 88100 Catanzaro, Italy; jessicafrangipane@gmail.com (J.F.); pasqualeviola@unicz.it (P.V.); alessiaastorina7@gmail.com (A.A.); chiarella@unicz.it (G.C.); 2 Department of Experimental and Clinical Medicine, Magna Graecia University, 88100 Catanzaro, Italy; 3 Radiology Unit, Dulbecco University Hospital, 88100 Catanzaro, Italy; 4 Department of Medicine, Surgery and Dentistry, University of Salerno, 84084 Salerno, Italy; ascarpa@unisa.it; 5 Otorhinolaryngology and Maxillo-Facial Surgery, Casa Sollievo della Sofferenza, 71013 San Giovanni Rotondo, Italy; teodoroaragona@yahoo.it; 6 Department of Otorhinolaryngology, Hannover Medical School, 30625 Hannover, Germany; emilioavallone@gmail.com; 7 ENT, Azienda Ospedaliero Universitaria delle Marche, 60126 Ancona, Italy; federicomaria.gioacchini@ospedaliriuniti.marche.it; 8 Department of Otolaryngology, Ospedale Fatebenefratelli Isola Tiberina—Gemelli Isola, 00186 Rome, Italy; dr.dlp@hotmail.it; 9 Department of Medicine & Surgery, Section of Otorhinolaryngology, University of Perugia, 06126 Perugia, Italy; giampietro.ricci@unipg.it (G.R.); gambacortavaleria@gmail.com (V.G.); 10 IRCCS Burlo Garofolo, Institute for Maternal and Child Health, 34137 Trieste, Italy; eva.orzan@burlo.trieste.it

**Keywords:** VHI, classroom, teachers, vocal effort

## Abstract

**Background/Objectives**: The voice is often perceived as a natural and spontaneous means of communication, but it involves complex interactions among physiological, psychological, and environmental factors. For teachers, whose profession relies heavily on vocal use, understanding and managing vocal strain is crucial. This study investigates the correlation between ambient noise levels in classrooms and teachers’ self-assessed voice-related quality of life, as measured by the Voice Handicap Index (VHI). The focus is on how classroom acoustics affect vocal health, considering the high incidence of vocal fatigue among educators. **Methods**: A pilot exploratory study was conducted from September 2022 to November 2022 involving four primary school teachers (two language and two science) from an Italian primary school. Classroom noise levels were recorded using the “Listen Responsibly” app at intervals during lessons. Following each lesson, teachers completed the VHI questionnaire to evaluate their voice-related quality of life. Statistical analyses included simple and multiple linear regressions, logistic regression, and Spearman’s correlation to assess the relationships between noise levels and VHI scores. **Results**: The study yielded 60 observations categorized into VHI Grade 1 (0–30) and Grade 2 (31–60). Significant differences were observed in average noise levels between these groups, with Grade 2 exhibiting higher noise levels (*p* < 0.0001). Simple and multiple linear regression analyses confirmed a positive correlation between average recorded noise and VHI scores, with each unit increase in noise associated with a 0.72 unit increase in VHI score (*p* < 0.0001). Logistic regression identified average recorded noise > 59.5 dB as a significant predictor of higher VHI grades (*p* < 0.0001). Spearman’s correlation confirmed a strong positive correlation (ρ = 0.77, *p* < 0.01). **Conclusions**: The study demonstrates a significant relationship between increased classroom noise levels and worse voice-related quality of life among teachers. These findings highlight the need for improved acoustic management in schools to reduce vocal strain. Implementing noise reduction strategies and enhancing classroom acoustics can help mitigate vocal health issues among educators, ultimately improving their professional and personal well-being.

## 1. Introduction

People often think of using their voice as something natural and spontaneous. However, it is not just a sound produced by a series of organs designed for this purpose; it is an expression of the person as a whole [1]. Various factors contribute to the voice, including individual characteristics, physiological and pathological aspects, and the potential for changes influenced by cultural, social, and work-related factors [2]. Therefore, a person’s vocal behavior can depend on their personality as well as their habits and the influence of their family and social environment [3]. Interestingly, one-third of working-age individuals have jobs where their voice is the primary tool, so it is crucial to understand and take care of it [4]. Given that the workplace environment plays a major role in vocal strain among teachers [5], we investigated how ambient classroom noise correlates with self-perceived voice handicap. The focus will be on teachers, who play a crucial role in the cultural development and growth of society. Teachers are responsible for disseminating information and conveying ideas, knowledge, and emotions, with their voice being their primary and essential tool [6]. As professionals, teachers are required to use their voices extensively and for prolonged periods, often in environments that expose them to various risk factors [6]. The incidence of vocal fatigue and related symptoms among teachers is notably high, leading to prolonged absences from work and sometimes necessitating surgical or rehabilitative interventions [7]. These issues carry not only economic consequences but also impact professional, personal, and emotional well-being. Effective communication between teachers and students is crucial in the school environment [7]. High levels of noise from inside or outside the school can significantly hinder a student’s learning, particularly in the early years, and can lead to serious vocal issues for teachers, who are often forced to strain their voices continuously [8,9]. The acoustic quality of school buildings and the subsequent well-being of both teachers and students are often among the most neglected aspects in school design and construction [10]. Noteworthily, the level of acoustic insulation from external noise hampers the clarity of teacher-student interactions through two main mechanisms: speech masking and reduced student attention [9,11,12,13].

The adverse effects of poor classroom acoustic conditions on teachers’ vocal health are well documented in the literature. Rantala and Sala demonstrated that acoustic parameters such as reverberation and speech transmission indices significantly influence vocal output, with noisier classrooms correlating with increased vocal strain despite paradoxically better listening conditions for students [14]. Moreover, Bottalico showed that teachers and other speakers adjust their speech in reverberant and noisy rooms by increasing vocal effort, pitch, and phonation time, all of which are risk factors for vocal fatigue [15]. This aligns with prior findings that excessive vocal load in noisy environments contributes to voice disorders [16]. Importantly, females—who make up the majority of teaching professionals—report greater vocal effort and vulnerability in background noise, as shown by Södersten et al. [17]. These findings underscore the need to further explore how the classroom environment impacts vocal health, particularly through low-cost, repeatable monitoring strategies. Noteworthily, a comprehensive scoping review by Mealings et al. examined 33 studies and found that excessive noise levels, large class sizes, and long reverberation times are frequently associated with increased vocal fatigue and psychological stress among teachers. Vocal health was the most commonly studied domain, with consistent evidence suggesting a detrimental impact of poor acoustic environments on teachers’ vocal function and overall well-being [18].

However, despite the well-established link between excessive classroom noise and risk of developing vocal disorders, there remains a lack of granular, real-world data capturing the day-to-day relationship between ambient noise and self-perceived vocal handicap in classroom settings. The aim of this study was to explore potential correlations between classroom noise levels and subjective vocal handicap, using the VHI as a proxy for teachers’ vocal discomfort, thereby contributing empirical evidence to this important occupational health issue.

## 2. Materials and Methods

### 2.1. Study Design

This pilot exploratory study is a single-center analysis of prospectively collected data from January 2022 to June 2022. The study aimed to evaluate the correlation between teachers’ perceived voice-related quality of life and the noise levels in primary school classrooms. Four primary school teachers—two teaching English and two teaching science—participated in the study. All four participating teachers were female. Data were gathered during lessons in first, second, and third-grade classrooms at an Italian primary school. All noise measurements were taken using the “Listen Responsibly” app installed on mid-range smartphones and tablets (Samsung Tab S6 Lite (Samsung Electronics, Suwon, Republic of Korea) and Google Pixel 4 (Google LLC, Mountain View, CA, USA)), with each device pre-tested in controlled acoustic conditions. While minor variability between models may exist, the previous literature supports acceptable consistency across such devices for comparative analysis [19]. Each teacher used the “Listen Responsibly” smartphone application (Amplifon company, Milan, Italy), functioning as a sound meter which records average noise levels in unweighted decibels (dB). The choice of using unweighted dB rather than frequency-weighted scales (such as dBA) was motivated by the aim to capture a broad-spectrum acoustic environment reflective of actual classroom noise, without frequency-dependent attenuation. The recordings were manually started by the teacher at three predefined time points during each 60 min lesson: at the 10th, 30th, and 50th minute. Each recording lasted 15 s, and during this time the teacher remained silent to prevent voice contamination in the noise sampling. The average of the three noise measurements taken during each lesson was used to calculate a single representative noise value, enabling direct comparison with the corresponding VHI score. The smartphone was consistently placed near the teacher’s desk, approximately 50–70 cm away from the teacher, ensuring standardized proximity across all measurements. To reduce the influence of external variables, measurements were carried out during similar time frames (mid-morning sessions), in comparable classroom sizes (ranging from 45 to 60 m^2^), and with teachers of similar seniority and workload. Although not all potential confounding factors were eliminated, efforts were made to ensure uniformity across observational contexts. Following each lesson, immediately after class dismissal, teachers completed the Vocal Handicap Index (VHI) questionnaire, consisting of 30 items where responses ranged from 0 (never) to 4 (always) [20] (see Supplemtary File S1). The VHI assesses self-reported voice-related quality of life across emotional, functional, and physical domains, with each domain comprising 10 items derived from patient interviews regarding a range of voice disorders [21]. Previous studies by Jacobson et al. have shown the VHI to possess good internal consistency, test–retest reliability, and correlation with patient-rated voice disorder severity [22]. This study utilized the Italian version of the VHI-30, validated by Schindler et al. [23]. Although the VHI was originally developed as a global measure of voice-related handicap, we used it here as a proxy for perceived vocal strain at the end of each lesson, consistent with the exploratory and repeated-measures design of this study. Prior to participation, teachers were instructed to respond to the VHI questionnaire with reference to the just-concluded lesson, rather than their general voice status.

Each teacher collected data weekly over five weeks, resulting in 15 observations per teacher across the three classrooms. Inclusion criteria required teachers to be certified by the Italian Ministry of Education for elementary school teaching, able to operate the “Listen Responsibly” app independently, and willing to participate. Exclusion criteria included language disorders, ear diseases, respiratory illnesses, mental disorders, recent ear or respiratory surgeries, fever, or acute infections. All participating teachers provided written informed consent. The research adhered to the ethical guidelines outlined in the Declaration of Helsinki.

### 2.2. Outcomes

The primary outcome is to examine the variation in average recorded noise levels across groups categorized by VHI scores: 0–30 (Grade 1), 31–60 (Grade 2), and 61–120 (Grade 3) [24]. Secondary objectives include identifying predictors of VHI scores, assessing the severity of VHI scores, and exploring the correlation between average classroom noise and VHI scores at the conclusion of each lesson.

### 2.3. Statistical Analysis

Data were managed in an Excel spreadsheet (Excel version 2016; Microsoft Inc., Redmond, WA, USA), and statistical analyses were conducted on a per-protocol basis using SPSS software (SPSS, version 22 for Windows; SPSS Inc., Chicago, IL, USA) and R/Software R Studio (R version 4.5.0). Normality of the data was assessed using the Kolmogorov–Smirnov and Shapiro–Wilk tests [25]. Categorical data are presented as frequencies (percentages) [26]. Normally distributed continuous data are expressed as mean ± standard deviation [27]. Non-normally distributed continuous data are presented as median (interquartile range) [28]. Statistical differences for normally distributed continuous data were evaluated using the unpaired Student’s *t*-test [29], while categorical and non-normally distributed continuous data were assessed using the chi-square test and Mann–Whitney test, respectively [30].

Simple and multiple linear regressions were performed to examine the relationship between classroom noise levels and teachers’ self-reported vocal handicap at the end of each lesson, assessed using VHI scores [31]. Logistic regression analysis was conducted to identify potential predictors for VHI grouping (with 80% of the dataset used for training and 20% for testing). Cutoff values were determined based on the median. Simple logistic regression models were used to predict class membership probability based on individual predictive variables. Spearman’s correlation test was employed to measure the correlation between average noise levels and VHI scores. A *p*-value < 0.05 was considered statistically significant for all analyses.

## 3. Results

Throughout the study period (January 2022–June 2022), a total of 60 observations were gathered. These observations were categorized based on the severity of VHI scores into three groups: VHI scores 0–30 (Grade 1), VHI scores 31–60 (Grade 2), and VHI scores 61–120 (Grade 3). Of these, 25 observations (42%) fell into the Grade 1 VHI category, while 35 observations (58%) fell into the Grade 2 VHI category. No Grade 3 VHI scores were recorded. There were no statistically significant differences between the Grade 1 and Grade 2 VHI groups concerning teachers (*p* = 0.60), teachers’ gender (*p* = 0.79), age (*p* = 0.2042), subject taught (*p* = 0.19), years of teaching experience (*p* = 0.2042), and classroom grades (*p* = 0.08). However, there was a significant difference in the number of students between the two groups (*p* = 0.0265).

**Figure 1:**
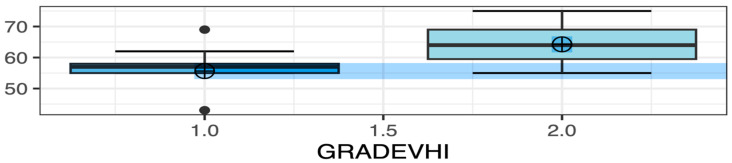
Box plots describing the average noise levels recorded between groups with VHI scores of grade 1 and grade 2.

The Grade 2 VHI group exhibited significantly higher average classroom noise levels compared to the Grade 1 VHI group (*p* < 0.0001—Figure 1). Additionally, noise levels recorded during the third recording were also significantly higher in the Grade 2 VHI group compared to Grade 1 (*p* = 0.0323). Detailed demographic data and group comparisons for VHI scores are presented in Table 1.

**Figure 2:**
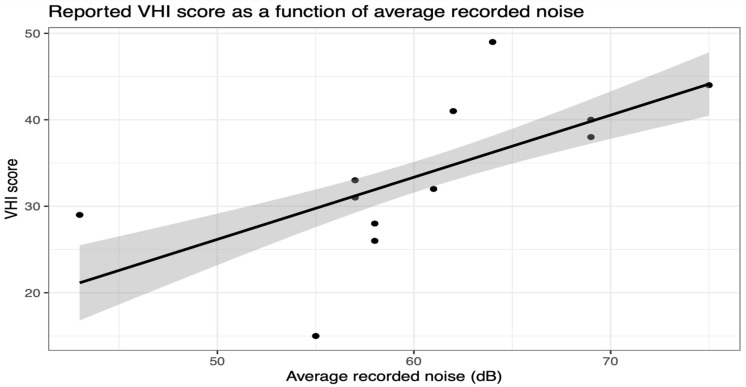
Data plotted with the linear regression line highlighting the positive relationship between the average recorded noise levels and the teachers’ self-assessed voice-related quality of life at the end of the lesson using the VHI score.

Simple linear regression analyses revealed a significant positive association between average recorded noise and VHI score (*p* < 0.0001) (Figure 2), as well as between noise measured during the initial assessment and VHI score (*p* = 0.0007), and between noise measured during the third assessment and VHI score (*p* = 0.0153). Each unit increase in average recorded noise was associated with a 0.72 unit increase in VHI score (±0.11). Notably, only average recorded noise showed a consistent positive relationship with VHI score in both simple and multiple linear regressions. Detailed findings are summarized in Table 2.

**Figure 3:**
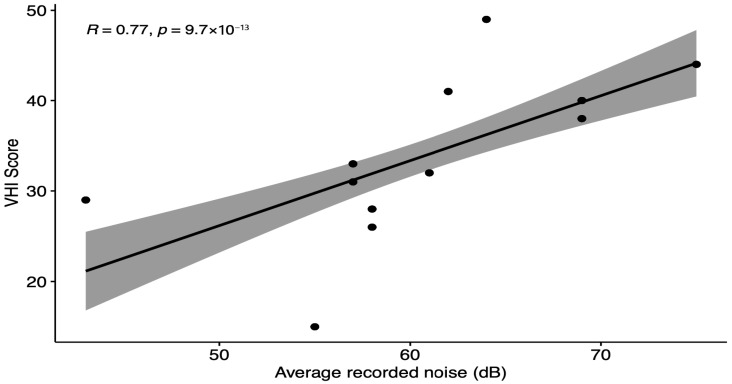
Data plotted using Spearman’s correlation method calculating the correlation between the average recorded noise levels and the VHI score.

Spearman’s correlation test confirmed a strong positive correlation between average recorded noise and VHI score, with a correlation coefficient of 0.77 and a highly significant *p*-value of 9.7 × 10^−13^ (Figure 3).

Simple logistic regression analysis showed that the number of students (*p* = 0.0313), average recorded noise (*p* = 0.0012), and average recorded noise > 59.5 dB (*p* < 0.0001) were significant individual predictors of the VHI group. It is worth noting that average recorded noise > 59.5 dB is associated with a mean increase of 2.72 in the log-odds of belonging to VHI Grade 2 group. The 59.5 dB cutoff value was selected based on the median average recorded noise. Additionally, a one-unit increase in average recorded noise is associated with a mean increase of 0.23 in the log-odds of belonging to VHI Grade 2 group. Detailed results are presented in Table 3.

## 4. Discussion

Teachers are among the at-risk groups prone to developing voice disorders due to extensive and prolonged use of their voice in noisy environments and inefficient phonation techniques [18]. This often results in functional dysphonia [32]. Vocal dysfunction can lead to extended sick leaves and the need for vocal rehabilitation, sometimes involving surgical interventions [33]. Besides impacting work performance negatively, it also affects quality of life, social interactions, and emotional well-being [34].

The aim of this study was to assess whether there is a correlation between perceived voice-related quality of life by teachers and the noise levels in school classrooms. The results of this work have shown clear findings, as illustrated in the tables presented above. Statistical analysis did not find significant differences between VHI Grade and the three classes examined, nor between VHI Grade and the group of participating teachers. Years of service and gender also did not show significant associations. Interestingly, classes with fewer students exhibited higher Grade 2 VHI results compared to Grade 1 VHI.

Teachers with longer service tenure did not experience greater vocal effort compared to those with less experience. Simple linear regression analysis indicated a positive relationship between average recorded noise and VHI score. An increase in classroom noise correlated with worse reported voice-related quality of life among teachers. The final measurement of classroom noise had the most significant impact on teachers’ VHI scores. Simple logistic regression analysis demonstrated that average recorded noise > 59.5 dB was a significant predictor of VHI Grade 2. Our findings complement earlier evidence that environmental noise leads to measurable vocal strain and increased self-perception of vocal impairment among teachers. The correlation between classroom noise and VHI scores—though limited by sample size—is consistent with physiological adjustments described by Bottalico, where increased vocal intensity and pitch were observed in response to reverberant environments [15]. Additionally, Summers et al. (1988) found that speech produced in noisy conditions not only changes acoustically but can paradoxically become more intelligible, possibly due to exaggerated articulatory effort—further increasing vocal loading [13]. Our results reaffirm long-standing concerns in occupational health. Vilkman (2004) emphasized that teachers represent a high-risk group for voice disorders due to both prolonged vocal use and environmental stressors like noise, poor acoustics, and poor indoor air quality [16]. Various preventive actions can mitigate vocal effort issues, including noise and reverberation control in classroom design, strategic school location in acoustically favorable areas, and tailored acoustical solutions for specific environmental needs [35]. Specifications for infrastructure purchases should include acoustic criteria, and existing school buildings could undergo acoustic improvements like installing sound-absorbing or isolating panels, optimizing furniture arrangement for reduced reverberation, and upgrading external wall insulation with soundproof panels [36]. While the Voice Handicap Index questionnaire is valuable for early vocal disorder detection, complementary measures such as acoustically adapting classrooms, promoting vocal hygiene, educating on respiratory dynamics, and fostering conscientious voice use are crucial in preventing vocal disorders from progressing to pathological stages. Establishing a standardized protocol tool for managing vocal issues before they escalate is essential [37]. Moreover, it is worth noting that all four participating teachers were female. This is relevant, as female sex is a known risk factor for voice disorders due to anatomical, hormonal, and occupational factors [38,39,40]. The homogeneity of sex among participants may have influenced the prevalence and severity of self-reported vocal symptoms in this sample.

Administrative and organizational interventions could involve installing voice amplification systems, situating noisy areas away from classrooms or towards exterior walls, employing teaching techniques that sustain high student engagement, reducing overcrowding, and adjusting teacher schedules to allow sufficient breaks between lessons [7]. Schools can support teachers by offering training on occupational hazards, organizing sessions on vocal hygiene and rest, teaching proper breathing techniques, relaxation methods, and maintaining good postural habits [41].

As defined by the World Health Organization, health is a multifaceted concept that encompasses physical, mental, and social well-being [42]. Consequently, the traditional assessment of a patient’s health, focusing solely on physical wellness, has transitioned to a more comprehensive approach that incorporates measures of quality of life [43]. Quality of life assessments for voice disorders include tools like the Voice Handicap Index (VHI), meeting criteria established by health agencies to gauge disability in speech disorders [44]. The 30-item VHI is easily administered, highly reproducible, and clinically robust. It serves as a valuable self-administered questionnaire for initial patient evaluations and subsequent follow-ups for those dealing with voice disorders [45]. The VHI provides clinics with supplementary insights into how patients perceive their voice-related quality of life and is strongly recommended in standard protocols for vocal assessments [45]. We believe the VHI questionnaire can be an effective tool for educators to detect and identify voice disorders early, prompting adjustments in behaviors that may exacerbate or chronically affect such issues. This underscores the importance of adequate training in vocal hygiene and education among teachers, enabling them to recognize the problem and adopt correct vocal techniques to prevent phonatory damage [46]. A prerequisite for the proper use of this tool is receiving sufficient training and information regarding the risks associated with vocal strain [46]. Given that a majority of the sample reported mild to moderate alterations, attention is drawn to the need for developing and implementing non-invasive, objective screening tools for functional vocal disorders [47]. This is crucial for the implementation of appropriate health surveillance programs by competent medical professionals and, more broadly, for adopting technical and structural solutions in classrooms to reduce the overall risk for teaching staff [48]. Suboptimal acoustics in classrooms are identified as the primary cause of reduced voice perception among teachers by students, thereby necessitating greater vocal effort and increasing the risk of vocal strain [49]. While measures such as vocal hygiene, education on respiratory dynamics, and conscientious use of the voice do not resolve these issues outright, they can help mitigate or prevent their progression to more severe pathological conditions [46].

Interestingly, our data showed that classrooms with fewer students had higher instances of Grade 2 VHI scores, indicating more severe perceived vocal handicap. This finding contrasts with previous literature, which typically associates larger class sizes with greater vocal strain due to increased vocal demand and higher ambient noise levels [18,50]. One possible explanation for our result may lie in contextual variables not captured by the current study, such as classroom layout, individual teacher speaking style, or behavioral issues in smaller groups requiring more vocal control. It is also possible that fewer students in certain settings led to a false sense of acoustic comfort, resulting in less conscious vocal modulation. Of note, our unexpected finding that smaller class sizes sometimes correlated with higher VHI scores could be interpreted in light of Lyberg-Åhlander et al. (2015), who showed that listener comprehension can degrade in adverse vocal conditions, such as when a speaker has a dysphonic voice [51]. It is possible that in quieter classrooms, the teacher’s perception of poor voice quality becomes more salient. Alternatively, the small sample size and potential confounders may have contributed to this apparent contradiction. This underscores the need for future research with larger samples and more granular control over contextual classroom variables.

Limitations of this study include the small sample size, the short-term study period, the potential variability introduced by different smartphone models, and the absence of complete control over classroom and pedagogical variables. These factors may influence the strength of associations observed and should be considered when interpreting the findings. A major limitation of this study is the small sample size, which included only four teachers. This constraint was due to the exploratory and pilot nature of the study, whose primary aim was to assess the feasibility of correlating real-time classroom noise data with self-perceived vocal effort. Although this restricts the statistical power and generalizability of the results, the repeated measurements and within-subject design provided useful initial evidence of potential associations. Future studies should aim to expand the sample size and include diverse teaching profiles and environments to validate and extend these findings. Noteworthily, the relatively low R values observed in the regression models reflect the complex and multifactorial nature of vocal effort and its relationship with environmental noise [52]. These values suggest that noise alone may not fully explain vocal strain, underlining the importance of integrating additional individual and contextual factors in future models. Another limitation of the present study is the use of the VHI to detect short-term vocal fluctuations. Although teachers were instructed to tailor their responses to each specific teaching session, the VHI was not originally designed for this purpose. Future work may benefit from incorporating alternative tools with higher temporal sensitivity, such as visual analog scales or ecological momentary assessments.

## 5. Conclusions

In conclusion, this pilot exploratory study establishes a significant correlation between ambient noise levels in classrooms and teachers’ voice-related quality of life, as measured by the Vocal Handicap Index. The findings highlight that higher noise levels are associated with increased vocal strain, underscoring the need for improved acoustic conditions in schools. To mitigate these risks, implementing preventive measures such as classroom acoustic adaptations, vocal hygiene education, and administrative support is essential to protect teachers’ vocal health and ensure effective communication in educational environments.

## Figures and Tables

**Table 1:** Comparison of demographic data and outcomes among VHI groups.

Variables	Group Voice Handicap Index = 1	Group Voice Handicap Index = 2	*p* Value
Number of observations	25 (42%)	35 (58%)	0.9522
Teacher (A/B/C/D)	5 (20%)/7 (28%)/8 (32%)/5 (20%)	10 (28.6%)/8 (22.8%)/7 (20%)/10 (28.6%)	0.60
Sex (M/F)	13 (52%)/12 (48%)	17 (48.5%)/18 (51.5%)	0.79
Subject (Science/English)	10 (40%)/15 (60%)	20 (57.2%)/15 (42.8%)	0.19
Class (1/2/3)	5 (20%)/12 (48%)/8 (32%)	15 (42.9%)/8 (22.9%)/12 (34.2%)	0.08
Age	33 (28–36)	33 (28–36)	0.2042
Years of service	12 (10–20)	10 (4–12)	0.2042
Number of pupils	28 (28–30)	28 (25–28)	0.0265
1st noise detection (dB)	55 (40–75)	66 (51.5–75)	0.2157
2nd noise detection (dB)	65 (56–68)	66 (59.5–71)	0.2941
3rd noise detection (dB)	60 (46–64)	68 (60–75)	0.0323
Average recorded noise (dB)	57 (55–58)	64 (59.5–69)	<0.0001

**Table 2:** Simple and multiple linear regression evaluating the relationship between recorded classroom noise levels and teachers’ self-assessed vocal handicap at the end of the lesson using the VHI score.

Predictors	Coeff.	Std. Err.	t Value	*p* > |t|
Average recorded noise (dB)	0.72/0.97	0.11/0.23	6.40/4.25	<0.0001/<0.0001
1st noise detection (dB)	0.23/0.06	0.06/0.08	3.57/0.78	0.0007/0.4385
2nd noise detection (dB)	0.13/−0.35	0.10/0.10	1.40/−3.63	0.168/0.0006
3rd noise detection (dB)	0.21/0.05	0.08/0.10	2.50/0.52	0.0153/0.6043

**Table 3:** Logistic regression analysis of predictive factors for the VHI group.

Predictors	Coeff.	Std. Err.	Z	*p* > |z|
Teacher (A/B/C/D)	−0.03	0.23	−0.12	0.9068
Sex (M/F)	0.14	0.52	0.26	0.7935
Subject (Science/English)	−0.69	0.53	−1.30	0.1928
Class (1/2/3)	−0.31	0.32	−0.96	0.3380
Age	−0.07	0.06	−1.24	0.2155
Years of service	−0.06	0.05	−1.20	0.2291
Number of pupils	−0.30	0.14	−2.15	0.0313
Average recorded noise	0.23	0.07	3.25	0.0012
Average recorded noise > 59.5 dB	2.72	0.69	4.07	<0.0001

## Data Availability

The data presented in this study are available on request from the corresponding author. The data are not publicly available due to privacy issues.

## Supplementary Materials

The following supporting information can be downloaded at: https://www.mdpi.com/article/10.3390/audiolres15050138/s1, File S1: Voice Handicap Index 30 Items.

## Abbreviations

The following abbreviations are used in this manuscript:

dB	Decibel
dBA	A-weighted decibel
VHI	Vocal Handicap Index
