# Supplementary material for: Teachers’ Voice-Related Quality of Life in Relation to Environmental Noise in Schools: A Multidimensional Study Using VHI Test and Listen Responsibly App
Source: Audiol Res. 2025 Oct 13;15(5):138. doi: 10.3390/audiolres15050138 (PMC12561122; doi:10.3390/audiolres15050138)
Supplement: Supplementary file 1 [file audiolres-15-00138-s001.zip › audiolres-3663320-supplementary.pdf]

audiology research

MDPI

Article

Teachers' Voice-related Quality of Life in Relation to Environmental Noise in Schools: A Multidimensional Study Using VHI Test and Listen Responsibly App

Jessica Frangipane[1], Pasquale Viola[1], Roberto Minici[2]*, Alfonso Scarpa[3], Alessia Astorina[1], Teodoro Aragona[4], Emilio Avallone[5], Federico Maria Gioacchini[6], Pietro De Luca[7], Giampietro Ricci[8], Valeria Gambacorta[8], Orzan Eva[9], Giuseppe Chiarella[1]

.

[1] Unit of Audiology, Regional Centre for Cochlear Implants and ENT Diseases, Department of Experimental and Clinical Medicine, Magna Graecia University, Catanzaro, Italy.
[2] Radiology Unit, Dulbecco University Hospital, Catanzaro, Italy.
[3] Department of Medicine, Surgery and Dentistry, University of Salerno, Salerno, Italy.
[4] Otorhinolaryngology and Maxillo-facial Surgery, Casa Sollievo della Sofferenza: San Giovanni Rotondo, IT
[5] Department of Otorhinolaryngology, Hannover Medical School, Hannover, Germany.
[6.] ENT, Azienda Ospedaliero Universitaria delle Marche, Ancona, Italy.
[7] Department of Otolaryngology, Ospedale Fatebenefratelli Isola Tiberina - Gemelli Isola, Rome, Italy.
[8] Department of Medicine & Surgery, Section of Otorhinolaryngology, University of Perugia, 06126 Perugia, Italy.
[9] IRCCS Burlo Garofolo, Institute for Maternal and Child Health, 34137 Trieste, Italy

* Correspondence

Academic Editor: Peter V. Paul

Received: 9 May 2025
Revised: 25 September 2025
Accepted: 11 October 2025
Published: 13 October 2025

Citation: Frangipane, J.; Viola, P.; Minici, R.; Scarpa, A.; Astorina, A.; Aragona, T.; Avallone, E.; Gioacchini, F.M.; De Luca, P.; Ricci, G.; et al. Teachers' Voice-related Quality of Life in Relation to Environmental Noise in Schools: A Multidimensional Study Using VHI Test and Listen Responsibly App. *Audiol. Res.* **2025**, *15*, x. https://doi.org/10.3390/xxxxx

Copyright: © 2025 by the authors. Submitted for possible open access publication under the terms and conditions of the Creative Commons Attribution (CC BY) license (https://creativecommons.org/licenses/by/4.0/).

Abstract: Background/Objectives: The voice is often perceived as a natural and spontaneous means of communication, but it involves complex interactions among physiological, psychological, and environmental factors. For teachers, whose profession relies heavily on vocal use, understanding and managing vocal strain is crucial. This study investigates the correlation between ambient noise levels in classrooms and teachers' self-assessed voice-related quality of life, as measured by the Voice Handicap Index (VHI). The focus is on how classroom acoustics affect vocal health, considering the high incidence of vocal fatigue among educators.

Methods: A pilot exploratory study was conducted from September 2022 to November 2022 involving four primary school teachers (two language and two science) from an Italian primary school. Classroom noise levels were recorded using the "Listen Responsibly" app at intervals during lessons. Following each lesson, teachers completed the VHI questionnaire to evaluate their voice-related quality of life. Statistical analyses included simple and multiple linear regressions, logistic regression, and Spearman's correlation to assess the relationships between noise levels and VHI scores.

Results: The study yielded 60 observations categorized into VHI Grade 1 (0-30) and Grade 2 (31-60). Significant differences were observed in average noise levels between these groups, with Grade 2 exhibiting higher noise levels ($p < 0.0001$). Simple and multiple linear regression analyses confirmed a positive correlation between average recorded noise and VHI scores, with each unit increase in noise associated with a 0.72 unit increase in VHI score ($p < 0.0001$). Logistic regression identified average recorded noise > 59.5 dB

as a significant predictor of higher VHI grades ($p < 0.0001$). Spearman's correlation confirmed a strong positive correlation (ϱ = 0.77, $p < 0.01$).

Conclusions: The study demonstrates a significant relationship between increased classroom noise levels and worse voice-related quality of life among teachers. These findings highlight the need for improved acoustic management in schools to reduce vocal strain. Implementing noise reduction strategies and enhancing classroom acoustics can help mitigate vocal health issues among educators, ultimately improving their professional and personal well-being.

Keywords: VHI, classroom, teachers, vocal effort

Supplementary File 1. Voice Handicap Index 30 Items

Voice Handicap Index – VHI-30

These are statements that many people have used to describe their voices & the effects of their voices on their lives. Circle the response that indicates how frequently you have the same experience.

0-never 1-almost never 2-sometimes 3-almost always 4-always

Part I-F

My voice makes it difficult for people to hear me.	0	1	2	3	4
People have difficulty understanding me in a noisy room.	0	1	2	3	4
My family has difficulty hearing me when I call them throughout the house.	0	1	2	3	4
I use the phone less often than I would like to.	0	1	2	3	4
I tend to avoid groups of people because of my voice.	0	1	2	3	4
I speak with friends, neighbors, or relatives less often because of my voice.	0	1	2	3	4
People ask me to repeat myself when speaking face-to-face.	0	1	2	3	4
My voice difficulties restrict my personal and social life.	0	1	2	3	4
I feel left out of conversations because of my voice.	0	1	2	3	4
My voice problem causes me to lose income.	0	1	2	3	4

SUBTOTAL

Part II-P

I run out of air when I talk.	0	1	2	3	4
The sound of my voice varies throughout the day.	0	1	2	3	4
People ask, "What's wrong with your voice?"	0	1	2	3	4
My voice sounds creaky and dry.	0	1	2	3	4
I feel as though I have to strain to produce voice.	0	1	2	3	4
The clarity of my voice is unpredictable.	0	1	2	3	4
I try to change my voice to sound different.	0	1	2	3	4
I use a great deal of effort to speak.	0	1	2	3	4
My voice is worse in the evening.	0	1	2	3	4
My voice "gives out" on me in the middle of speaking.	0	1	2	3	4

SUBTOTAL

Part III-E

I am tense when talking to others because of my voice.	0	1	2	3	4
People seem irritated with my voice.	0	1	2	3	4
I find other people don't understand my voice problem.	0	1	2	3	4
My voice problem upsets me.	0	1	2	3	4
I am less outgoing because of my voice problem.	0	1	2	3	4
My voice makes me feels handicapped.	0	1	2	3	4
I feel annoyed when people ask me to repeat.	0	1	2	3	4
I feel embarrassed when people ask me to repeat.	0	1	2	3	4
My voice makes me feel incompetent.	0	1	2	3	4
I am ashamed of my voice problem.	0	1	2	3	4

SUBTOTAL

TOTAL______
